# Plant Extracts and Natural Compounds for the Treatment of Urinary Tract Infections in Women: Mechanisms, Efficacy, and Therapeutic Potential

**DOI:** 10.3390/cimb47080591

**Published:** 2025-07-25

**Authors:** Ya-Ting Hsu, Hsien-Chang Wu, Chung-Che Tsai, Yao-Chou Tsai, Chan-Yen Kuo

**Affiliations:** 1 School of Post-Baccalaureate Chinese Medicine, Tzu Chi University, Hualien 970374, Taiwan; sophie761122@hotmail.com (Y.-T.H.); xuang@tzuchi.com.tw (H.-C.W.); 2 Department of Chinese Medicine, Taipei Tzu Chi Hospital, The Buddhist Tzu Chi Medical Foundation, No. 289, Jianguo Rd., Xindian Dist., New Taipei City 23142, Taiwan; 3 Department of Research, Taipei Tzu Chi Hospital, The Buddhist Tzu Chi Medical Foundation, New Taipei City 23142, Taiwan; chungche.tsai@gmail.com; 4 Department of Nursing, Cardinal Tien College of Healthcare and Management, New Taipei City 231038, Taiwan; 5 Division of Urology, Department of Surgery, Taipei Tzuchi Hospital, The Buddhist Tzu Chi Medical Foundation, New Taipei City 23142, Taiwan; 6 School of Medicine, Buddhist Tzu Chi University, Hualien 970374, Taiwan; 7 Institute of Oral Medicine and Materials, College of Medicine, Tzu Chi University, Hualien 970374, Taiwan

**Keywords:** urinary tract infection, recurrent urinary tract infection, lower urinary tract dysfunction, medicinal plants, natural compounds

## Abstract

Urinary tract infections (UTIs) are among the most prevalent bacterial infections in women, with high recurrence rates and growing concerns over antimicrobial resistance. The need for alternative or adjunctive therapies has spurred interest in plant-based treatments, which offer antimicrobial, anti-inflammatory, antioxidant, and immune-modulatory benefits. This review summarizes the mechanisms of action, clinical efficacy, and therapeutic potential of various medicinal plants and natural compounds for preventing and treating UTIs in women. Notable candidates include cranberry, bearberry, pomegranate, green tea, and other phytochemicals with proven anti-adhesive and biofilm-disrupting properties. Evidence from clinical trials and meta-analyses supports the role of cranberry natural products and traditional herbal medicines (THMs) in reducing UTI recurrence, especially when combined with antibiotics. Notably, A-type proanthocyanidins in cranberry and arbutin in bearberry are key bioactive compounds that exhibit potent anti-adhesive and biofilm-disrupting properties, offering promising adjunctive strategies for preventing recurrent urinary tract infections. Additionally, emerging therapies, such as platelet-rich plasma (PRP), show promise in restoring bladder function and reducing infection in women with lower urinary tract dysfunction. Overall, plant-based strategies represent a valuable and well-tolerated complement to conventional therapies and warrant further investigation through high-quality clinical trials to validate their efficacy, safety, and role in personalized UTI management.

## 1. Introduction

Urinary tract infections (UTIs) predominantly affect women, with over 50% experiencing at least one episode in their lifetime [1]. The incidence of UTIs is particularly high in sexually active women, postmenopausal women, and those with underlying conditions, such as diabetes or urinary catheterization [2]. Recurrent UTIs (rUTIs) are a common issue, with approximately 20–30% of women experiencing a second infection within six months of the initial episode [3]. The increasing prevalence of antibiotic-resistant uropathogens, such as *Escherichia coli* (*E. coli*), *Klebsiella pneumoniae* (*K. pneumoniae*), and *Proteus mirabilis* (*P. mirabilis*), further complicates UTI management and underscores the urgent need for alternative or adjunctive therapeutic approaches [4].

Plant extracts have gained attention as potential therapeutic agents for UTIs due to their rich bioactive compound content, including phenolics, flavonoids, tannins, alkaloids, and essential oils [5]. These compounds exert antimicrobial, anti-inflammatory, antioxidant, and immune-modulatory effects, targeting various stages of UTI pathogenesis, from bacterial adhesion and biofilm formation to inflammation and oxidative stress [6,7]. Furthermore, plant-based therapies present a lower risk of adverse effects and antibiotic resistance compared to conventional antibiotics, making them attractive candidates for the long-term management of rUTIs [8].

This review examines the therapeutic role of plant extracts in UTI treatment, focusing on well-studied medicinal plants, including cranberry (*Vaccinium macrocarpon*, *V. macrocarpon*), garlic (*Allium sativum*, *A. sativum*), oregano (*Origanum vulgare*, *O. vulgare*), pomegranate (*Punica granatum*, *P. granatum*), and green tea (*Camellia sinensis*, *C. sinensis*). The mechanisms of action, clinical efficacy, and potential integration of these plant extracts into existing treatment protocols are discussed, with particular emphasis on their role in mitigating antibiotic resistance and enhancing patient outcomes.

## 2. Pathogenesis and Risk Factors of Urinary Tract Infections in Women

Urinary tract infections predominantly affect women due to anatomical and physiological factors that facilitate bacterial colonization and invasion [9]. The shorter urethra, proximity of the urethral opening to the anus, and hormonal changes during menstruation, pregnancy, and menopause increase the risk of UTIs in women [10].

The majority of UTIs are caused by uropathogenic *E. coli* (UPEC), which possess virulence factors, such as adhesins, fimbriae, and toxins, that enable them to adhere to and invade uroepithelial cells [11]. Other common pathogens include *K. pneumoniae*, *P. mirabilis*, *Staphylococcus saprophyticus* (*S. saprophyticus*), *Enterococcus* spp., and *Candida albicans* (*C. albicans*) [12]. These bacteria employ multiple strategies to establish infection, including biofilm formation, immune evasion, and resistance to host defenses [13]. In addition to these common bacterial pathogens, certain viruses and protozoa can also contribute to UTIs, especially in immunocompromised individuals. BK virus and adenovirus are associated with hemorrhagic cystitis, particularly after kidney or stem cell transplantation. Herpes simplex virus type 2 may cause urethral inflammation and dysuria during primary infection. Among protozoa, *Trichomonas vaginalis* is a sexually transmitted parasite that can infect the urethra and mimic UTI symptoms. In endemic regions, *Schistosoma haematobium* may cause chronic bladder inflammation and hematuria. Although less frequent than bacterial UTIs, these non-bacterial pathogens warrant attention in specific clinical settings.

Biofilm formation is a critical factor in recurrent UTIs (rUTIs), as bacterial biofilms shield pathogens from immune responses and antibiotic treatment [14]. Biofilms are structured communities of bacteria encased in a protective extracellular matrix composed of polysaccharides, proteins, and nucleic acids [15]. Once established, biofilms can persist on uroepithelial surfaces, urinary catheters, and other medical devices, contributing to chronic infections and an increased risk of recurrence [12]. Hormonal fluctuations, particularly during menopause, can alter the vaginal microbiota and decrease levels of protective lactobacilli, resulting in increased colonization by uropathogens [16]. Additionally, sexual activity, use of spermicides, and diaphragm use can disrupt the vaginal flora, further predisposing women to UTIs [17].

rUTIs are defined as three or more episodes of infection within 12 months or two or more episodes within six months [18]. rUTIs are associated with persistent bacterial reservoirs in the bladder, including intracellular bacterial communities, and uropathogens that evade host defenses by residing in quiescent, latent states [19]. Therefore, understanding these pathogenic mechanisms provides a rationale for exploring plant extracts with anti-adhesive, biofilm-disrupting, and antimicrobial properties as potential therapeutic agents for the prevention and treatment of UTIs in women.

## 3. Natural Therapeutic Strategies for UTIs: Comparative Overview and Mechanistic Insights of Medicinal Plants

The clinical burden of recurrent and drug-resistant UTIs in women has renewed interest in plant-derived prophylactics and adjuvants. Twenty-four botanicals with demonstrable activity against uropathogens were classified into three phytochemical clusters—berry-derived, leaf/herb-derived, and seed/root/resin-derived (Table 1). The following overview integrates recent mechanistic data and human-dose considerations.

### 3.1. Berry-Derived Botanicals

Cranberry (*V. macrocarpon*) is the most extensively studied phytotherapy for UTIs. Its fruits are rich in polyphenols, particularly A-type PACs (MIC = 0.5–112 mg/mL), which inhibit Escherichia coli adhesion to urothelial cells via P and type 1 fimbriae, disrupt early biofilm formation, and downregulate quorum-sensing pathways [41]. Gut microbiota-mediated biotransformation enhances PAC bioactivity [42]. Co-administration of D-mannose, which targets the FimH adhesin, provides complementary anti-adhesion effects [43,44]. Together, cranberry PACs and D-mannose offer a non-antibiotic strategy for preventing UTIs in women with recurrent infections, pediatric patients, and individuals undergoing urological procedures.

Cranberries also contain flavonoids, anthocyanins, phenolic acids, and triterpenoids, which contribute antioxidant, anti-inflammatory, antimicrobial, and immunomodulatory activities [45]. Among these, A-type PACs are considered key bioactives due to their ability to inhibit both antibiotic-sensitive and resistant *E. coli* strains [46]. Meta-analyses suggest a 24% reduction in UTI risk with a daily intake of ≥36 mg PACs, though variability in extract standardization and limited trial sizes highlight the need for further phase III studies [47]. Amid rising antimicrobial resistance, interest in alternatives such as natural products, antimicrobial peptides, and nanotechnology is increasing. Flavonoids, a major class of polyphenols, show broad-spectrum anti-infective activity through membrane disruption, inhibition of nucleic acid synthesis, and enzyme interference [48,49]. Cranberry and D-mannose supplements have shown moderate efficacy in reducing UTI incidence, particularly in high-risk groups [50]. However, current clinical data are limited by methodological inconsistencies and small sample sizes. Further well-designed, large-scale clinical trials are essential to validate efficacy and inform standardized treatment protocols (Table 2).

Bearberry (*A. uva-ursi*) leaf provides arbutin, which is hydrolysed in alkaline urine to hydroquinone, yielding MIC values of 0.21 to 0.6 mg/mL against *E. coli* and *S. saprophyticus* [53,54]. A daily dose equivalent to 420 mg anhydrous arbutin releases less than 11 µg/kg body weight of hydroquinone, which remains well below toxicological thresholds and supports its short-term use in acute cystitis [54] (Table 2). Combined clinical experience with cranberry confirms broad-spectrum coverage and good tolerability [55].

Pomegranate (*P. granatum* L.), originally from South Asia and now cultivated in tropical and subtropical regions like Mexico, is valued for its rich flavor and functional properties. It is widely used in products such as jellies, jams, and beverages, and is recognized for its health benefits, including effectiveness against diseases and pathogenic microorganisms [56]. A previous study underscored the potent antibacterial properties of *P. granatum* leaf extract and identified epicatechin, kaempferol, and apigenin as promising compounds for the development of novel therapeutic agents targeting multidrug-resistant (MDR) *E. coli* [57]. Among the medicinal plants tested, the fruit peels of *P. granatum* demonstrated the strongest antibacterial activity against UTI-causing bacteria [58]. Another study demonstrated that aqueous pomegranate peel extract demonstrates promising antimicrobial and anti-virulence activity against UTIs caused by *E. coli* [59]. *P. granatum* sarcotesta lectin, a natural lectin from pomegranate fruit, has also been found to exert promising antibacterial and anti-biofilm properties against MDR *E. coli*, particularly when used in combination with β-lactam antibiotics [60]. In summary, *P. granatum* is a potent natural source of antibacterial agents effective against MDR *E. coli*. Its bioactive compounds and extracts, especially from peel and sarcotesta, hold significant potential for the development of novel therapies to combat antibiotic-resistant infections, particularly UTIs.

Other berry-derived botanicals, including lingonberry, roselle, pomegranate, and juniper berry, offer additional therapeutic potential. Bioactive compounds such as condensed tannins from *V. vitis-idaea*, hibiscus acid from Hibiscus sabdariffa, ellagitannins and lectins from *P. granatum*, and α-pinene from *J. communis* contribute to diverse mechanisms of action, including iron chelation, membrane permeabilization, and lectin-mediated biofilm inhibition [56,57,58,59,60]. Extracts from *P. granatum* peel and sarcotesta have demonstrated activity against MDR *E. coli* and show synergistic effects with β-lactam antibiotics, reducing the fractional inhibitory concentration index to ≤0.5 [57,60].

### 3.2. Leaf- and Herb-Derived Botanicals

Green tea (*C. sinensis*) is rich in polyphenols, particularly flavanols (catechins) such as (−)-epigallocatechin-3-gallate (EGCG), (−)-epicatechin (EC), (−)-epicatechin-3-gallate (ECG), and (−)-epigallocatechin (EGC) [55]. Among these, EGCG and EGC have demonstrated notable antimicrobial activity. EGCG has been shown to inhibit bacterial cell division by targeting FtsZ-dependent septation and to disrupt redox homeostasis. However, due to differences in solubility and metabolism, only the more water-soluble EGC is consistently detected in the urine of individuals with UTIs [61]. Although the precise mechanism by which green tea exerts its effects on UTIs remains unclear, preliminary studies suggest that daily catechin intakes of 200–400 mg may reduce asymptomatic bacteriuria. Nonetheless, well-designed dose-finding trials are still needed, particularly in populations with recurrent UTIs.

Herbs that produce essential oils, such as thyme, oregano, and rosemary, contain active compounds including thymol, carvacrol, and rosmarinic acid. These constituents can collapse bacterial membrane potential and suppress fimH transcription at sub-inhibitory concentrations, with thymol exhibiting a MIC of approximately 2 µg/mL. However, issues such as volatility and mucosal irritation have posed formulation challenges, leading to the development of microencapsulation techniques and intravesical gel prototypes currently under preclinical investigation.

In addition, several diuretic and anti-inflammatory herbs, including *S. virgaurea* (goldenrod), *P. crispum* (parsley, a source of apigenin), and *U. dioica* (stinging nettle), have been reported to increase urine output and reduce proinflammatory cytokine release [62,63,64]. Notably, extracts from *S. virgaurea* have demonstrated the ability to reduce *E. coli* biofilm formation. However, they also antagonized the post-antibiotic effects of amikacin and ciprofloxacin, highlighting the importance of combination testing before clinical application [65]. A comparative summary of key leaf- and herb-derived botanicals is provided in Table 3, including their major phytochemicals, proposed mechanisms, therapeutic effects in UTI management, and known limitations or safety concerns. This overview helps to contextualize both the traditional use and modern pharmacological evaluation of these agents.

### 3.3. Seed-, Root- and Resin-Derived Botanicals

Liposterolic extracts from pumpkin seed (*C. pepo*) and saw palmetto (*S. repens*) have been shown to modulate androgen receptor activity and muscarinic signaling [68,69]. These effects help improve bladder emptying and may indirectly reduce the risk of urinary tract infections. Saw palmetto also demonstrates antimicrobial activity, with a MIC ranging from 1.5 to 2.1 mg/mL [33].

Roots rich in organosulfur and polyphenolic compounds, such as garlic (which contains allicin), ginger (a source of gingerols), and turmeric (which provides curcumin), exert antimicrobial effects by targeting thiol-containing enzymes, DNA gyrase, and oxidative stress pathways. Notably, curcumin nanoparticles have been reported to enhance the efficacy of fluoroquinolones against MDR bacterial isolates.

Medicinal herbs such as licorice, myrrh, and barberry (which is rich in berberine) exhibit broad-spectrum antimicrobial properties, including antibacterial, antifungal, and antiviral activity. These may be particularly beneficial against non-bacterial uropathogens. Additionally, echinacea contributes primarily through its immunomodulatory effects, potentially supporting host defense in urinary tract infections.

### 3.4. Comparison of Antibacterial Activity and Clinical Potential of Plant Extracts Versus Conventional Antibiotics in Urinary Tract Infections

Conventional antibiotics such as ciprofloxacin and ampicillin exhibit very low MICs against key uropathogens, including *E. coli* and *K. pneumoniae*, typically ranging from 0.001 to 0.06 mg/mL [70,71]. In contrast, most plant extracts such as cranberry, bearberry, green tea polyphenols, and willow bark show higher MICs, generally between 0.063 and 10 mg/mL [72,73]. For example, cranberry proanthocyanidins and green tea EGCG demonstrate antibacterial activity against *E. coli* with MICs of 250 μg/mL and less than 4 mg/mL, respectively [61,74,75], although these values are still higher than those of standard antibiotics. Nonetheless, several plant extracts have shown synergistic effects when combined with antibiotics, especially against MDR strains [76]. Such combinations can enhance antibiotic efficacy, reduce required dosages, and help mitigate the development of resistance [77]. For instance, the lowest MIC values were observed when tigecycline was combined with fermented cranberry juice against *Staphylococcus aureus* strains, followed by the same combination against *Enterobacter cloacae* strains [77]. Additionally, plant extracts often present favorable safety profiles and low toxicity, making them suitable for adjunctive or preventive therapy, especially in recurrent or uncomplicated UTI cases [78].

### 3.5. Mechanisms of Plant Extracts in Disrupting Bacterial Biofilms and Their Role in Recurrent UTI Management

Bacterial biofilms play a crucial role in the pathogenesis and UTIs, particularly those involving UPEC. Biofilms are structured communities of bacteria embedded in a self-produced extracellular polymeric substance (EPS), which protects them from host immunity and antibiotic treatment, contributing to persistent and recurrent infections [12]. Plant extracts, rich in phytochemicals such as polyphenols, flavonoids, terpenoids, and alkaloids, have been shown to interfere with multiple stages of biofilm development [79]. Certain phytochemicals, such as cranberry-derived proanthocyanidins and garlic-derived allicin, can impair bacterial adhesion to uroepithelial cells and abiotic surfaces by downregulating fimbrial adhesins like type 1 and P fimbriae, thus preventing the initial step of biofilm formation [80,81]. In addition, many plant-derived compounds interfere with quorum sensing (QS), the bacterial communication system that regulates biofilm maturation. Compounds such as cinnamaldehyde and curcumin have been shown to inhibit QS signaling pathways in *E. coli* and *Pseudomonas aeruginosa*, leading to reduced production of EPS and virulence factors [82]. Furthermore, certain enzymes and secondary metabolites found in plant extracts are capable of degrading key components of the EPS matrix, such as polysaccharides and proteins, thereby promoting biofilm disruption and increasing bacterial susceptibility to antibiotics [83]. Notably, plant extracts also exhibit synergistic effects with antibiotics by enhancing bacterial membrane permeability and inhibiting efflux pumps within biofilm-embedded cells, ultimately facilitating improved antibiotic penetration and efficacy [84]. Therefore, by targeting these mechanisms, plant extracts offer a promising adjunctive approach to reduce biofilm-associated antibiotic resistance and recurrence rates in UTIs. Their multi-target activity and lower propensity to induce resistance make them suitable for long-term or prophylactic use in patients with rUTIs.

## 4. Herbal and Emerging Therapies for Urinary Tract and Lower Urinary Tract Symptoms: Clinical Evidence and Non-Antibiotic Strategies

Herbal medicines, particularly cranberry products, show clear benefits in preventing rUTIs in women [85]. Clinical evidence supports the use of saw palmetto, stinging nettle root, and pumpkin seed extracts for male lower urinary tract symptoms (LUTS) related to benign prostatic hyperplasia (BPH), while European golden rod and combination herbal preparations show some evidence for managing UTIs [86]. This systematic review and meta-analysis evaluated the effectiveness of cranberry-containing products in preventing UTIs. Results indicated these products appear to offer a moderate protective effect against UTIs, particularly in women and children [87]. Moreover, the use of dietary supplements, particularly saw palmetto berry extract, has become common in the US for managing LUTS in men, often related to BPH. While early research suggested saw palmetto may offer modest clinical benefits by targeting BPH-related mechanisms, recent studies have shown inconsistent results, leaving its effectiveness uncertain. Short-term use appears safe, though long-term safety data are lacking [88]. A meta-analysis evaluated those traditional herbal medicines (THMs) used alongside antibiotics and identified strong supportive benefits in the treatment and prevention of rUTIs. While THMs alone are less effective than antibiotics, they may still serve as a potential alternative therapy, especially for patients seeking non-antibiotic options [89]. In addition to antimicrobial and anti-adhesive properties, several plant extracts exhibit immunomodulatory effects that further contribute to their therapeutic potential in urinary tract infections. *P. granatum* (pomegranate) peel extract and its major ellagitannin components, including punicalagin, punicalin, and ellagic acid, have been reported to suppress pro-inflammatory cytokines such as tumor necrosis factor alpha, interleukin (IL)-6, and IL-8 while inhibiting Th1 and Th17 immune responses. At lower concentrations, pomegranate-derived compounds enhance IL-10 production, a cytokine associated with regulatory T cells, indicating their potential to restore immune balance and reduce recurrence. Similarly, catechins from *C. inensis* (green tea), particularly EGCG, modulate immune activity by attenuating oxidative stress and downregulating inflammatory mediators such as cyclooxygenase-2 and inducible nitric oxide synthase. These immunoregulatory effects support mucosal healing and may help prevent recurrent infections by reinforcing local immune defenses. Liao et al. reported that PRP therapy shows promise as an innovative, non-antibiotic treatment for rUTIs in women with lower urinary tract dysfunction (LUTD), potentially improving bladder health and reducing recurrence [90]. Taken together, herbal therapies and emerging treatments like PRP offer valuable non-antibiotic strategies for managing UTIs and LUTS. Cranberry products and THMs used with antibiotics show strong preventive effects for rUTIs, while saw palmetto and other herbal supplements may benefit male LUTS (Table 4).

Several clinical studies have evaluated the efficacy of THMs as adjuncts to antibiotic therapy in the prevention of rUTIs. Herbal formulations containing *V. macrocarpon* (Cranberry), *A. uva-ursi* (bearberry), and *Angelica sinensis*, among others, have shown significant synergistic effects when used alongside antibiotics. For example, a randomized controlled trial by Beerepoot et al. demonstrated that cranberry capsules reduced rUTI recurrence comparably to trimethoprim, with fewer side effects and without promoting antibiotic resistance [91]. Similarly, results showed that the combination of Sanjin tablets with either gatifloxacin or levofloxacin significantly improved cure rates, total effective rates, and bacterial clearance compared to antibiotic treatment alone. Additionally, the combination therapy was associated with a lower recurrence rate and no serious adverse events were reported. However, the overall quality of evidence was rated as low according to Grading of Recommendations, Assessment, Development and Evaluations criteria [92] as well as other [93]. These outcomes are thought to result from THMs’ ability to inhibit bacterial adhesion, modulate inflammation, and restore urogenital microbial balance. Importantly, the combination therapy also reduced the overall need for long-term antibiotic use, suggesting a role for THMs in antibiotic stewardship. While more large-scale trials are warranted, current evidence supports the adjunctive use of THMs as a promising strategy for rUTI prevention.

While plant-based therapies are widely regarded as safe alternatives or adjuncts to conventional antibiotics for preventing recurrent UTIs, their long-term use particularly in women with underlying conditions such as diabetes mellitus or chronic catheterization warrants careful consideration [94]. Some herbal agents, such as *A. uva-ursi* (bearberry), contain hydroquinone derivatives like arbutin, which may pose a risk of hepatotoxicity or nephrotoxicity if used in excessive or prolonged doses [54]. In diabetic patients, interactions with hypoglycemic medications should be considered, as certain plant extracts including berberine and cranberry may influence glucose metabolism [95,96]. Additionally, immunocompromised or catheterized patients may be more susceptible to unintended microbial shifts or allergic reactions associated with herbal compounds. Despite these risks, most clinical studies report minimal adverse effects when plant-based treatments are used at therapeutic doses and for limited durations [97,98]. Nevertheless, individualized assessment and physician oversight are essential when using these therapies in high-risk populations. Further pharmacovigilance and large-scale safety studies are needed to better characterize the long-term safety profile of these agents.

Unlike conventional antibiotics, which typically target a single bacterial pathway or protein, plant-based treatments often consist of complex mixtures of bioactive compounds that exert multifaceted antimicrobial effects, including disruption of bacterial membranes, inhibition of quorum sensing, suppression of biofilm formation, and interference with bacterial metabolism. These multi-target mechanisms reduce the selective pressure that typically drives resistance development [99]. Moreover, many plant-derived agents, such as polyphenols, flavonoids, and tannins, modulate the host immune response and oxidative stress, providing indirect antimicrobial benefits that do not solely rely on bacterial killing [100]. This dual action directs antibacterial activity, and immune modulation makes it more difficult for bacteria to develop resistance. Furthermore, studies have shown that certain phytochemicals can restore antibiotic sensitivity in resistant strains by inhibiting efflux pumps or disrupting biofilms, thereby enhancing the efficacy of existing antibiotics [101,102]. Therefore, plant-based therapies represent a promising strategy in combating UTIs while potentially reducing the emergence and spread of antimicrobial resistance.

Oxidative stress plays a critical role in the pathogenesis of UTIs [103]. During infection, uropathogenic bacteria such as *E. coli* trigger the host immune response, leading to the generation of excessive reactive oxygen species (ROS) by neutrophils and macrophages as part of the inflammatory cascade [104]. Activation of the NF-E2-related factor 2 (NRF2) pathway has been shown to occur in response to ROS generated by UPEC. In urothelial cells, NRF2 activation reduces ROS levels, suppresses inflammation and cell death, and promotes the expulsion of UPEC, thereby decreasing the bacterial burden [105]. Although ROS are crucial for bacterial clearance, excessive ROS production can lead to collateral damage of epithelial cells, compromise tight junctions, and disrupt mucosal integrity, ultimately aggravating tissue injury and contributing to chronic inflammation or recurrent infections [106]. Plant-derived extracts are rich in natural antioxidants such as flavonoids, polyphenols, tannins, and phenolic acids, which scavenge free radicals and inhibit lipid peroxidation [107,108]. Previous studies demonstrated that chlorogenic acid (from bearberry) and curcumin (from turmeric) modulate oxidative signaling pathways and reduce pro-inflammatory cytokine expression [109,110]. By alleviating oxidative stress, plant-derived antioxidants help preserve uroepithelial integrity, promote mucosal repair, limit bacterial colonization, and may thereby reduce the likelihood of recurrent urinary tract infections.

Plant-based therapies for UTIs are generally more cost-effective and accessible than conventional antibiotics, particularly in low- and middle-income countries [111]. Many medicinal plants, such as *P. granatum* (pomegranate), *C. sinensis* (green tea), *U. dioica* (nettle), and *V. macrocarpon* (cranberry), are widely cultivated, locally available, and inexpensive to process into teas, extracts, or capsules. Unlike synthetic antibiotics, which often require prescription access, cold-chain storage, and strict dosing protocols, plant-derived preparations can be produced and distributed with lower infrastructure costs [112,113,114].

## 5. Discussion

Recent evidence suggests that combining multiple plant extracts or their bioactive constituents may offer synergistic therapeutic effects against urinary tract infections by simultaneously targeting distinct aspects of pathogenesis [8]. For example, cranberry-derived PACs inhibit *E. coli* adhesion, while pomegranate ellagitannins modulate pro-inflammatory cytokines and green tea catechins suppress oxidative stress and quorum sensing pathways [41,115]. When used in combination, these compounds may provide enhanced biofilm inhibition, immune modulation, and microbial clearance, as their mechanisms of action are complementary. Moreover, studies have shown that combinations of punicalagin, punicalin, and ellagic acid from pomegranate enhance anti-inflammatory responses, while maintaining non-cytotoxic profiles. Similarly, combinations of bearberry and cranberry have demonstrated additive or synergistic effects in suppressing bacterial growth and adhesion in vitro [116]. Such plant-based synergies may also reduce the required doses of individual compounds, minimize toxicity and improve tolerability. While preclinical evidence is encouraging, further high-quality studies are needed to systematically evaluate optimal combinations, dosing strategies, and potential interactions to guide the clinical application of synergistic phytotherapy in UTI management [42].

Although numerous studies have demonstrated the short-term benefits of plant-based therapies such as cranberry, bearberry, green tea, and pomegranate extracts in preventing rUTIs [42,61,91], however, their long-term safety and efficacy remain insufficiently characterized, especially in vulnerable populations like postmenopausal women or patients with diabetes [52]. These individuals often exhibit altered immune responses, hormonal imbalances, or underlying comorbidities that may influence treatment outcomes [117,118]. Therefore, longitudinal clinical trials with extended follow-up periods are essential to evaluate sustained therapeutic effects, potential cumulative toxicity, and real-world adherence. Such studies would also help identify subgroups that may benefit most from phytotherapeutic interventions and inform personalized prevention strategies for rUTIs.

Personalized UTI management is an emerging paradigm that considers individual variability in host microbiota, immune responses, and antimicrobial resistance profiles [119]. Recent advances in microbiome sequencing and resistance gene profiling have made it feasible to identify patient-specific patterns that influence treatment outcomes [120]. In this context, plant-based therapies offer a promising adjunct to antibiotics, as their multi-target mechanisms can be tailored to specific microbial and host factors [121]. For instance, patients with high abundance of UPEC strains expressing type 1 fimbriae may particularly benefit from cranberry-derived proanthocyanidins, which inhibit bacterial adhesion [122]. Similarly, green tea catechins may be especially useful in individuals with oxidative-stress-associated inflammation due to their potent antioxidant and immunomodulatory effects [123]. Moreover, using microbiota analysis to assess the impact of herbal compounds on beneficial versus pathogenic bacteria could help avoid dysbiosis and support long-term urinary tract health [124]. Future clinical research should focus on integrating phytotherapeutic strategies into personalized UTI care, guided by microbiome and resistance profiling.

## 6. Summary

UTIs, particularly recurrent ones, are a significant health concern for women worldwide due to their high prevalence, impact on quality of life, and the growing challenge of antimicrobial resistance. While antibiotics remain the standard treatment, their overuse has led to resistant strains of uropathogens, necessitating alternative therapeutic approaches. This review highlights the promising role of plant extracts and natural compounds in the prevention and treatment of UTIs in women.

Cranberry-derived PACs, bearberry arbutin, green tea catechins, and pomegranate polyphenols have been found to exert anti-adhesive, antimicrobial, anti-inflammatory, and biofilm-inhibitory properties. Clinical evidence supports the efficacy of cranberry products and THMs, especially when used alongside conventional antibiotics, in reducing UTI recurrence. Moreover, emerging therapies such as PRP injections show potential in managing rUTIs associated with LUTD by enhancing urothelial repair.

This review primarily focuses on the biological mechanisms, therapeutic potential, and current evidence supporting the use of plant-based therapies in the prevention and treatment of UTIs. However, it does not provide a detailed analysis of clinical trial methodologies, including aspects such as population diversity, placebo controls, and the standardization of outcome measures, such as UTI recurrence rates and symptom resolution criteria. These factors are critical for ensuring the reliability and generalizability of clinical findings but fall outside the scope of this manuscript. Future studies should aim to address these methodological considerations to strengthen the clinical evidence base and facilitate the integration of phytotherapeutics into standard UTI management protocols.

Together, these findings underscore the therapeutic potential of plant-based interventions and novel non-antibiotic strategies for managing UTIs in women. However, standardized formulations, optimized dosages, and well-designed clinical trials are essential to validate their efficacy and integrate them into evidence-based practice.

## 7. Conclusions

Recurrent UTIs in women present a significant clinical challenge, particularly in the face of rising antibiotic resistance and limited long-term treatment options. This review highlights the growing body of evidence supporting the use of plant extracts and natural compounds, including cranberry, bearberry, green tea, pomegranate, and various traditional herbal formulations, as promising adjuncts or alternatives to conventional therapies. These botanicals exhibit a wide range of bioactivities, including antimicrobial, anti-inflammatory, diuretic, anti-adhesive, and biofilm-disrupting effects, which target key mechanisms involved in UTI pathogenesis and recurrence. While some plant-based therapies, especially cranberry products and THMs, have demonstrated clinical efficacy in preventing UTIs, others show potential that merits further investigation.

Emerging approaches, such as PRP therapy, may further expand the scope of non-antibiotic treatments, particularly for patients with underlying LUTD. However, the variability in extract composition, limited standardization, and gaps in large-scale clinical evidence call for more rigorous, well-designed studies. Future research should aim to validate efficacy, ensure safety, explore synergistic combinations, and identify biomarkers for personalized treatment. Integrating plant-based and emerging therapies into UTI management protocols could reduce reliance on antibiotics and improve outcomes for women with recurrent infections.

Despite growing scientific evidence supporting the efficacy of plant-based therapies in preventing and managing urinary tract infections, these approaches remain underutilized in clinical settings. Raising awareness among healthcare professionals and the general public through education, clinical guidelines, and patient counseling is crucial to promote informed use of phytotherapeutic strategies. Such efforts can help integrate evidence-based plant-derived interventions into mainstream UTI management, especially in the context of rising antibiotic resistance.

## Figures and Tables

**Table 1:** Medicinal Plants Used for UTI.

Common Name (Scientific Name)	Used Part	Key Active Compounds	Minimum Inhibitory Concentration (MIC, mg/mL)	Common Formulations	References
Cranberry (*V. macrocarpon*)	Fruit	A-type proanthocyanidins (PACs)	0.5–112	Juice and capsule	[20]
Lingonberry (*Vaccinium vitis-idaea*, *V. vitis-idaea*)	Leaf/Fruit	PACs, tannins	0.24–0.48 mg/mL (*E. coli*) 125 mg/mL (*C. albicans*)	Concentrate and tea	[21]
Bearberry (*Arctostaphylos uva-ursi*, *A. uva-ursi*)	Leaf	Arbutin	0.21–0.6 (*Cutibacterium acnes*)	Tea and capsule	[22]
Roselle (*Hibiscus sabdariffa*)	Calyx	Hibiscus acid, anthocyanins	7–10 (*E. coli*); 25–50 (multidrug-resistant *Acinetobacter baumannii*)	Acid tea and powder	[23]
Pomegranate (*P. granatum*)	Peel	Punicalagin	0.78–6.25 (*E. coli* and antibiotic-resistant Gram-positive bacteria)	Peel extract	[24]
Juniper berry (*Juniperus communis*, *J. communis*)	Berry (oil)	α-pinene	6.25 (*E. coli* and *Staphylococcus aureus*)	Essential oil and tincture	[25]
Green tea (*C. sinensis*)	Leaf	EGCG	3.25–50 (*E. coli*)	Tea and powder	[26]
Thyme (*Thymus vulgaris*)	Leaf (oil)	Thymol	12.5–50 (*E. coli*)	Essential oil and tincture	[27]
Oregano (*O. vulgare*)	Leaf (oil)	Carvacrol	0.256 (*E. coli*)	Essential oil	[28]
Rosemary (*Rosmarinus officinalis*)	Leaf	Rosmarinic acid	12.5–50 (*E. coli*)	Extract and essential oil	[27]
Stinging nettle (*Urtica dioica*, *U. dioica*)	Leaf	Chlorogenic acid	0.195 (*E. coli*)	Tea and powder	[29]
Goldenrod (*Solidago virgaurea*, *S. virgaurea*)	Aerial parts	Diterpenes	1.5 (*E. coli*)	Extract and tea	[30]
Java tea (*Orthosiphon stamineus*)	Leaf	Sinensetin, rosmarinic acid	0.31–2 (*E. coli*)	Tea and extract	[30]
Parsley (*Petroselinum crispum*, *P*. *crispum*)	Leaf/seed oil	Apiol	0.35–0.4 (*P. aeruginosa*); >70 (Gram-positive bacteria)	Essential oil and tincture	[31]
Pumpkin seed (*Cucurbita pepo*, *C*. *pepo*)	Seed oil	Δ7-sterols	≥10 (*E. coli*)	Oil capsule	[32]
Saw palmetto (*Serenoa repens*, *S*. *repens*)	Fruit (oil)	Fatty acids	1.5–2.1 (*E. coli*)	CO_2_ oil and softgel	[33]
Garlic (*A. sativum*)	Bulb	Allicin	375 (*E. coli*)	Raw, oil, and tablet	[34]
Ginger (*Zingiber officinale*)	Rhizome	Gingerols	100 (*E. coli*)	Powder and tincture	[35]
Turmeric (*Curcuma longa*)	Rhizome	Curcumin	0.25–0.5 (*E. coli*)	Powder and capsule	[36]
Licorice (*Glycyrrhiza glabra*)	Root	Glycyrrhizin	12.5 (*E. coli* and *Klebsiella* spp.)	Extract and tablet	[37]
Myrrh (*Commiphora myrrha*)	Resin	Furano-sesquiterpenes	15.6 (*E. coli* and *C. albicans*)	Tincture and essential oil	[38]
Barberry (*Berberis vulgaris*)	Root/peel	Berberine	40 (*E. coli*)	Tincture and capsule	[39]
Echinacea (*Echinacea purpurea*)	Aerial parts	Cichoric acid	>5 (*E. coli* and *C. albicans*)	Extract and tablet	[40]

**Table 2:** Natural therapeutics for UTIs covering mechanisms and clinical efficacy.

Natural Product	Key Bioactive Compounds	Mechanism of Action	Clinical Effects	References
Cranberry	Flavonoids, phenolic acids, anthocyanins, triterpenoids, A-type PACs	- Inhibits *E. coli* adhesion to urothelial cells - Disrupts biofilm formation - Antioxidant, anti-inflammatory, antimicrobial, immunomodulatory effects	- Reduces UTI incidence, especially in women and children - Effective against both antibiotic-susceptible and resistant *E. coli*	[42,45,46,51,52]
D-Mannose	Monosaccharide sugar	- Binds to *E. coli* type 1 fimbriae to prevent adhesion to the urothelium	- Inhibits bacterial attachment - Shows potential in combination with cranberry products”	[44,50]
Cranberry + D-Mannose	PACs + monosaccharide sugar	- Synergistic anti-adhesive effects against uropathogenic *E. coli*	- Promising non-antibiotic strategy for recurrent UTI prevention in susceptible populations	[44,50]
Bearberry	Arbutin, hydroquinone (HQ)	- Antiseptic activity via release of hydroquinone in urine - Diuretic effect aids bacterial flushing	- Broad-spectrum antimicrobial activity - Used in traditional medicine for UTI treatment”	[53,54,55]

**Table 3:** Leaf- and herb-derived botanicals for UTI management with key compounds, mechanisms, therapeutic roles, and safety notes.

Plant/Botanical	Key Bioactive Compounds	Mechanism of Action	Therapeutic Role in UTIs	Notes/Concerns	References
Goldenrod (*S. virgaurea*)	Flavonoids, saponins, phenolic acids	Diuretic; reduces bacterial survival and biofilm formation	Promotes urinary flushing; reduces bacterial persistence	May weaken the efficacy of certain antibiotics (e.g., amikacin and ciprofloxacin)	[62,65]
Lovage (*Levisticum officinale*)	Phthalides, coumarins	Diuretic and mild antiseptic	Aids in pathogen clearance through increased urine output	Use with caution in renal disorders	[62]
Parsley (*P. crispum*)	Apigenin	Diuretic and anti-inflammatory	Reduces inflammation; supports flushing of pathogens	High doses may interact with diuretics or anticoagulants	[62,63]
Stinging nettle (*U. dioica*)	Flavonoids, phenolic acids	Diuretic, anti-inflammatory, coagulant	Supports detoxification and urinary tract cleansing; reduces inflammation	Generally safe at recommended doses	[62,64]
Cranberry (*V. macrocarpon*)	A-type PACs	Anti-adhesive against *E. coli*, antimicrobial, antioxidant	Prevents bacterial adhesion to urothelium; reduces risk of recurrent UTIs	Interactions with warfarin reported in rare cases	[66]
Bearberry (*A. uva-ursi*)	Arbutin (HQ)	Antiseptic and diuretic	Kills uropathogens in urine; traditionally used for bladder infections	Use with caution in long-term treatment due to potential HQ toxicity	[66]
Juniper (*J. communis*)	Essential oils, flavonoids	Diuretic, antimicrobial	Increases urine flow; inhibits bacterial growth	May irritate kidneys with prolonged use	[66]
Goldenseal (*Hydrastis canadensis*)	Berberine	Inhibits bacterial adhesion and biofilm formation	Potential use in preventing UTI-causing bacterial colonization	Use cautiously in pregnancy; can interact with liver enzymes	[67]
Oregon grape (*Mahonia aquifolium*)	Berberine, alkaloids	Similar to goldenseal; antimicrobial, anti-adhesive	May prevent bacterial colonization and support UTI management	Similar precautions as with goldenseal	[67]

**Table 4:** Herbal medicines and emerging therapies for UTIs and LUTS: clinical evidence and applications.

Therapy/Product	Target Condition	Mechanism/Effect	Clinical Evidence/Benefits	References
Cranberry (*V. macrocarpon*)	Recurrent UTIs (particularly in women and children)	Inhibits *E. coli* adhesion; anti-adhesive, antimicrobial, antioxidant	Moderate preventive effect in susceptible populations	[85,87]
Saw palmetto (*S. repens*)	Male LUTS due to BPH	Anti-androgenic and anti-inflammatory; modulates prostatic growth pathways	Modest benefits reported; recent studies show inconsistent results; short-term use appears safe	[86,88]
Stinging nettle (*U. dioica*) root	Male LUTS	Anti-inflammatory and mild diuretic effects	Evidence supports use in combination with saw palmetto for BPH-related LUTS	[86]
Pumpkin seed extract	Male LUTS	Anti-inflammatory, modulates bladder function	Shown to improve urinary flow and reduce symptoms in men with BPH	[86]
European goldenrod (*S. virgaurea*)	UTIs	Diuretic and antimicrobial; reduces bacterial survival and biofilm formation	Used traditionally; some evidence supports use in combination therapies	[86]
Combination THMs + Antibiotics	Recurrent UTIs	Enhances antimicrobial efficacy; targets multiple infection pathways	Meta-analysis shows significantly improved outcomes vs. antibiotics alone	[89]
Traditional Herbal Medicines (alone)	Recurrent UTIs	Varies depending on formula; includes anti-inflammatory and antimicrobial effects	Less effective than antibiotics alone but may serve as non-antibiotic alternatives	[89]
PRP	rUTIs with LUTD in women	Regenerates urothelial lining, anti-inflammatory, enhances barrier function	Promising results in reducing recurrence and improving bladder symptom	[90]

## Data Availability

A literature search was conducted using the PubMed bibliographic database.

## Abbreviations

The following abbreviations are used in this manuscript:

BPH	Benign prostatic hyperplasia
CCR	Clinical cure rate
CNKI	China National Knowledge Infrastructure
EC	Epicatechin
ECG	Epicatechin-3-gallate
EGC	Epigallocatechin
EGCG	Epigallocatechin-3-gallate
EOT	End-of-treatment
EPS	Extracellular polymeric substance
HQ	Hydroquinone
IL	Interleukin
LUTD	Lower urinary tract dysfunction
LUTS	Lower urinary tract symptoms
MIC	Minimum inhibitory concentration
NRF2	NF-E2-related factor 2
PACs	Proanthocyanidins
PAE	Post-antibiotic effect
PRP	Platelet-rich plasma
QS	Quorum sensing
RR	Risk ratio
rUTIs	Recurrent urinary tract infections
THMs	Traditional herbal medicines
TSA	Trial sequential analysis
UPEC	Uropathogenic *Escherichia coli*
UTI	Urinary tract infection
